# Predictive diagnostic and/or prognostic biomarkers obtained from routine blood biochemistry in patients with solitary intracranial tumor

**DOI:** 10.5937/jomb0-24722

**Published:** 2021-01-26

**Authors:** Ulas Yuksel, Mustafa Ogden, Alemiddin Ozdemir, Ucler Kisa, Bulent Bakar

**Affiliations:** 1 Kirikkale University, Faculty of Medicine, Department of Neurosurgery, Yahsihan, Kirikkale, Turkey; 2 Kirikkale University, Faculty of Medicine, Department of Biochemistry, Kirikkale, Turkey

**Keywords:** brain metastasis, C-reactive protein, erythrocyte sedimentation rate, glioma, intracranial tumor, prognosis, prognoza, intrakranijalni tumor, gliom, sedimetacija eritrocita, C-reaktivni protein, metastaze mozga

## Abstract

**Background:**

Radiological and/or laboratory tests may be sometimes inadequate distinguishing glioblastoma from metastatic brain tumors. The aim of this study was to find possible predictive biomarkers produced from routine blood biochemistry analysis results evaluated preoperatively in each patient with solitary brain tumor in distinguishing glioblastoma from metastatic brain tumors as well as revealing short-term prognosis.

**Methods:**

Patients admitted to neurosurgery clinic between January 2015 and September 2018 were included in this study and they were divided into GLIOMA (n=12) and METASTASIS (n=17) groups. Patients' data consisted of age, gender, Glasgow Coma Scale scores, duration of stay in hospital, Glasgow Outcome Scale (GOS) scores and histopathological examination reports, hemoglobin level, leukocyte, neutrophil, lymphocyte, monocyte, eosinophil, basophil and platelet count results, neutrophil-lymphocyte ratio and platelet-lymphocyte ratio values, C-reactive protein (CRP) and erythrocyte sedimentation rate (ESR) levels were evaluated preoperatively.

**Results:**

The CRP levels of METASTASIS group (143.10 mg/L) were higher than those of GLIOMA group (23.90 mg/L); and it was 82% sensitive and 75% specific in distinguishing metastatic brain tumor from glioblastoma if CRP value was >55.00 mg/L. A positive correlation was determined between GOS score and hemoglobin level and between ESR and CRP values. However, GOS scores were negatively correlated with the ESR level and duration of stay in hospital.

**Conclusions:**

Study results demonstrated that CRP values could be predictive biomarker in distinguishing metastatic brain tumor from glioblastoma. In addition, ESR, CRP, hemoglobin levels and duration of stay in hospital could be prognostic biomarkers in predicting short-term prognosis of patients with solitary brain tumor.

## Abstract (sr)

**Uvod:**

Radiološki i/ili laboratorijski testovi mogu ponekad da budu neadekvatni za otkrivanje glioblastoma u metastatskih tumora mozga. Svrha ovog ispitivanja bila je da se pronađu mogući prediktivni biomarkeri iz rutinskih biohemijskih analiza krvi koji su procenjivani preoperativno kod svakog pacijenta sa solitarnim tumorom mozga pri razdvajanju glioblastoma od metastatskih tumora mozga kao i pri proceni kratkotrajne prognoze.

**Metode:**

Pacijenti koji su bili primljeni na neurohiruršku kliniku između januara 2015 i septembra 2018 uključeni su u ovo proučavanje i bili su podeljeni u GRUPE GLIOMA (n= 12) i METASTAZE (N = 17). Podaci o pacijentima obuhvatili su starost, pol, Glasgow Coma skor skalu (GOS), dužinu boravka u bolnici i histopatološko ispitivanje, nivo hemoglobina, leukocita, neutrofila, monocita, eozinofila, bazofila, broja trombocita, odnos neutrofila-limfocita, vrednosti odnosa trombociti-limfociti, C-reaktivni protein (CRP) i sedimentaciju eritrocita (ESR) koji su procenjivani preoperativno.

**Rezultati:**

Nivoi CRP su bili viši u grupi sa metastazom (143,10 mg/L) nego u grupi sa gliomom (23,90 mg/L; ako su vrednosti bile veće od 55,00 mg/L CRP je bio 82% osetljiviji i 75% specifičniji za razdvajanje metastaza tumora mozga od glioblastoma. Utvrđena je pozitivna korelacija između GOS skora i nivoa hemoglobina i između ESR i CRP vrednosti. Međutim, GOS skorovi su bili u negativnoj kore laciji sa ESR nivoom i dužinom ostanka u bolnici.

**Zaključak:**

Rezultati izučavanja su pokazali da vrednosti CRP mogu biti prediktivni marker pri razdvajanju metastatskog tumora mozga od glioblastoma. Takođe, nivoi ESR, CRP, hemoglobina i dužina ostanka u bolnici mogu biti prediktivni prognostički biomarkeri za kratkotrajnu prognozu kod pacijenta sa solitarnim tumorom mozga.

## Introduction

Preoperative diagnosis of primary glial tumors and metastatic brain tumors is still a problem today [1]. Currently, reliable differentiation and diagnosis of glioblastoma from brain metastasis in radiological imaging may be important to make a decision on the tumor staging, surgical approach and medical treatment modalities [2] [3] [4]. Previous history of primary malignancy, the presence of most of the lesions on the gray-white junction and the absence of infiltration on radiological images are traditionally used to help differentiate metastases from glioblastoma. However, on conventional magnetic resonance (MR) images, brain metastasis and glioblastoma may appear to resemble and even primary brain malignancies may develop during the presence of the systemic cancer, and/or solitary intracranial brain metastasis may occur as the first sign of extracranial malignancy [1].

On the other hand, it is accepted that if the brain tumor in the patient is metastatic tumor, the primary tumor has to be detected in the body using radiologic or other imaging methods and treated appropriately. Therefore, being able to predict that the tumor in the brain may be a metastatic tumor would enable these treatment options to be considered and performed. On the other hand, if it is possible to predict that this tumor is glial tumor, the radiation that the patient would receive during the general body scan would also decrease [5] [6]. Recently, to solve these problems mentioned above and to support and increase the diagnostic power of radiological examinations, many studies have described many molecular markers classifying the tumor and predicting the patient survival. Unfortunately, most of those markers have been provided from the tumor tissue [7].

Currently, to additional advanced biomarkers for diagnosis, rating, classification and prognosis of glioblastoma are still under investigation. However, there are still very few studies on serum-based biomarkers that can be easily obtained with a minimally invasive sample technique diagnosing intracranial tumors and estimating the treatment response [7] [8].

Therefore, the aim of this study was to find possible predictive biomarkers produced from routine blood biochemistry analysis results evaluated preoperatively in each patient with solitary brain tumor in distinguishing glioblastoma from metastatic brain tumors as well as revealing short-term prognosis. Furthermore, in this study it was aimed to be able to support and reinforce the results of MR or computerized tomography (CT) scan which are accepted as conventional diagnostic methods by using these biomarkers.

## Materials and Methods

### Participants

This study was carried out after approval from the Clinical Studies Local Ethics Committee.

**Figure 1:**
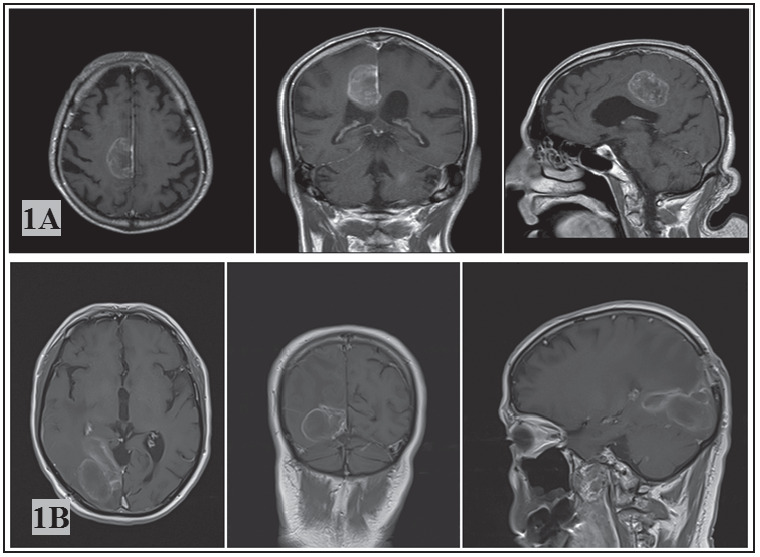
Cranial MR images of patients with intracranial solitary tumor: (1A) axial, coronal and sagittal T1 weighted images of MR with gadolinium in patient with glioblastoma; (1B) axial, coronal and sagittal T1 weighted images of MR with gadolinium in patient with brain metastasis

In this study, patients with solitary intracranial tumor diagnosed with glioblastoma or metastatic brain tumor histopathologically who admitted to the neurosurgery clinic between January 2015 and June 2018 were investigated retrospectively (Figure 1). Patients treated with recurrence of intracranial tumor, patients treated performing radiosurgery, patients with multiple intracranial tumors and paediatric participants (age<16 years) were ruled out from the study.

Participants included in this study were separated into two groups mentioned below:

GLIOMA group which was consisted of 12 patients who had glioblastoma.

METASTASIS group which contained 17 patients who had solitary brain metastasis.

### Methods

Age, gender, length of stay in hospital, Glasgow Coma Scale (GCS) scores, histopathological examination reports and Glasgow Outcome Scale (GOS) scores were recorded. The hemoglobin (Hb) and erythrocyte sedimentation rate levels, leukocyte count results, neutrophil count results, lymphocyte, monocyte, eosiophil, basophil and platelet count results, neutrophil to lymphocyte ratio (NLR) and platelet to lymphocyte ratio results (PLR) were examined. Moreover, C-reactive protein (CRP), serum glucose, sodium (Na), potassium (K), aspartate aminotransferase (AST), alanine aminotransferase (ALT), creatinine and blood urea nitrogen (BUN) levels were investigated.

The neurological status of the participants was examined using the scales described at below:

Glasgow Coma Scale (GCS): It was used to examine and describe the patient's consciousness level and neurological status at admission to the hospital [9].

Glasgow Outcome Scale (GOS): It is used to describe the participants' neurological status and consciousness level after their discharged from the hospital [10].

### Biochemical evaluation

Biochemical parameters which were used in this study were obtained preoperatively from the venous blood samples of the patients when they admitted to the neurosurgery clinic.

Serum glucose (reference interval 4.11-6.05 mmol/L), sodium (reference 136-146 mmol/L), potassium (reference interval 3.5-5.1 mmol/L), creatinine (reference interval 74.26-109.62 µmol/L), and BUN (reference interval 6.07-15.35 mmol/L), ALT (reference interval 0.08-0.68 µkat/L), AST (reference interval 0.08-0.67 µkat/L) and CRP (reference interval 1.5-50.00 mg/L) level values were measured by using an analyzer device (Roche Diagnostic COBAS c501). The hemoglobin level (reference interval 100.00-180.00 g/L), platelet (reference interval 150.00-500.00 ×10^9^/L), leukocyte (reference interval 4.40-11.30 ×10^9^/L), neutrophil (reference interval 1.10-9.60 ×10^9^/L), lymphocyte (reference interval 0.50-6.00 ×10^9^/L), eosinophil (reference interval 0-1.00 ×10^9^/L), basophil (refer-ence interval 0-0.30 ×10^9^/L) and monocyte (reference interval 0.10-1.40 ×10^9^/L) count values were measured by using an analyzer device (Mindray BC-6800, Shenzen, China). Erythrocyte sedimentation rate (ESR) value (reference interval <20 mm/hour) was measured by automated system (ESR 40, Sistat Diagnostics).

### Statistical analysis

Statistical Packages for the Social Sciences (SPSS version 20, IBM, USA) software was used to be performed the statistical analysis test. G power analysis (G*Power, version 3.1.9.2) was used to be tested whether the numbers of the patients included in the study were sufficient to apply statistical analysis tests to the results obtained.

The *Mann-Whitney U* test was used to compare the non-parametric data of the groups (p<0.05). The *Independent Samples* *t* test was used to compare the parametric data of the groups (p<0.05). To explore the predictive properties of the parameters the *ROC-Curve* test was performed and the sensitivity and specificity ratios were determined by setting »cutoff« values. To explore the correlation between the study parameters, *Spearman’s rho Correlation* test was performed (p<0.05). The *Logistic Regression* test and *Likelihood Ratio* test were applied to the variables for the prediction of the »best« diagnostic and/or prognostic variable, respectively (p<0.05).

## Results

A total of 29 patients (female = 7, male = 22) with a mean age of 60 ± 11.23 were included in this study. The histopathological examinations revealed that 12 patients had solitary gliablastoma, 17 had solitary metastatic tumors (6 patients had adenocarcinoma, 9 patients had malignant epithelial tumor, 2 patients had round cell tumor). G power analysis showed that to generalize the findings of this study, the minimum sample size of the METASTASIS group should be 11 and minimum sample size of GLIOMA group should be 11 (effect size d = 1.453, actual power = 0.950).

Age, gender, GCS and GOS score values were not different between the two groups. The leukocyte, neutrophil, lymphocyte, monocyte, eosinophil, basophil and platelet count results which were within the reference interval value were not different between the groups. Furthermore, the similarity was found between the groups in terms of the ESR, NLR and PLR values. In addition, serum glucose, sodium, potassium, ALT, AST, BUN and creatinine results were similar between the groups. There was no difference in the length of hospital stay between the groups. However, CRP level values were significantly different between the two groups (Z = -3.410, p = 0.001). The CRP level values of the METASTASIS group (143.10 mg/L) were higher than GLIOMA group values (23.90 mg/L) (Table 1).

**Table 1:** Table shows the differences between the groups in terms of demographic findings and biochemistry results. The Independent Samples t test, Mann-Whitney U test and Chi-Square test (p<0.05). (*) t value; (‡) Pearson Chi-square value. N: number of subjects, Min: minimum, Max: maximum, SD: standart deviation, t: t value, Z: Z score, X2: Pearson Chi-Square value, ESR: Erytrocyte sedimentation rate, AST: Aspartate aminotransferase, ALT: Alanine aminotransferase

		GLIOMA	METASTASIS		
Variable		Mean±SD/Median (min-max)/N (%)	Mean±SD/Median (min-max)/N (%)	t/Z/X2	p
Age (year)		59.50±13.20	61.71±9.94	-0.514*	0.612
Gender	Female	5 (17.2%)	2 (6.9%)	3.435‡	0.064
	Male	7 (24.1%)	15 (51.7%)		
Glasgow Coma Scale		15 (12–15)	15 (9–15)	-1.191	0.234
Hemoglobin level (g/L)		134.1±1.17	138.1±2.02	-0.605*	0.550
Leukocyte count (×10^9^/L)		10.48±2.46	10.05±4.26	0.313*	0.757
Neutrophil count (×10^9^/L)		7.84 (3.89–10.89)	6.67 (2.06–20.48)	-0.443	0.658
Lymphocyte count (×10^9^/L)		2.27±1717.99	1.82±872.50	0.935*	0.358
Monocyte count (×10^9^/L)		0.48±285.65	0.50±382.02	-0.621*	0.540
Eosinophil count (×10^9^/L)		0.07 (0.01–0.29)	0.07 (0.00–0.27)	-0.155	0.877
Basophil count (×10^9^/L)		0.03 (0.01–0.08)	0.03 (0.01–0.19)	-0.266	0.790
Platelet count (×10^9^/L)		222.50±50.86	232.59±113.01	-0.288*	0.775
Neutrophil-lymphocyte ratio		4.07 (1.47–31.75)	4.19 (1.65–12.82)	-0.664	0.506
Platelet-lymphocyte ratio		17.69±15.77	18.04±14.72	-0.061*	0.952
ESR (mm/hour)		15.50 (5–69)	41 (4–86)	-1.308	0.191
C-reactive protein (mg/L)		23.90 (3.00–167.90)	143.10 (22.40–870.00)	-3.410	0.001
Glucose (mmol/L)		7.08 (4.72–10.88)	7.10 (4.38–14.32)	-0.288	0.773
Sodium (mmol/L)		138 (134–148)	138 (132–143)	-0.781	0.435
Potassium (mmol/L)		4.36±0.58	4.39±0.57	-0.155*	0.878
AST (µkat/L)		0.32 (0.23–1.29)	0.25 (0.15–0.65)	-1.269	0.205
ALT (µkat/L)		0.37 (0.13–1.72)	0.28 (0.12–0.83)	-0.421	0.673
Creatinin (µmol/L)		61.76±0.29	71.67±0.17	-1.520*	0.140
Blood urea nitrogen (mmol/L)		14.34±13.13	16.41±16.91	-0.997*	0.328
Duration of hospital stay (day)		11.50 (4–35)	11 (6–144)	-0.155	0.877
Glasgow Outcome Scale		5 (1–5)	5 (3–5)	-1.114	0.265

**Figure 2:**
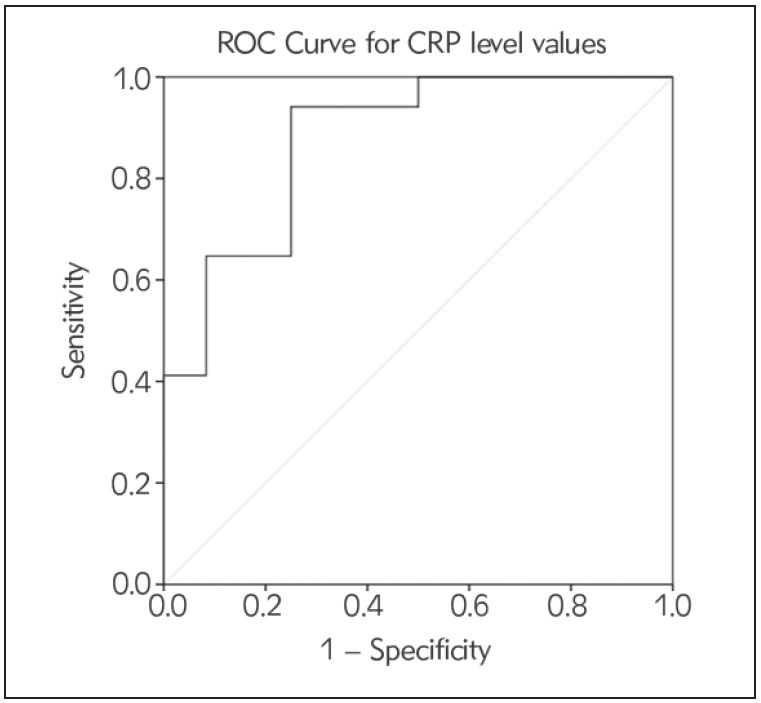
The ROC-Curve analysis results of the C-reactive protein level values

The *ROC-Curve* test demonstrated that if the CRP level value was measured greater than 55.00 mg/L, it could be 82% sensitive and 75% specific in distinguishing the solitary brain metastatis from the glioblastoma (area = 0.877, p = 0.001, cut-off value = 55.00 mg/L) (Table 2, Figure 2). The *Logistic Regression* test revealed that CRP value could be the best laboratory parameter in distinguishing the solitary brain metastasis from the glioblastoma (B = 0.202, Wald = 4.353, p = 0.037).

**Table 2:** Table demonstrates the results of the ROC-Curve test for the C-reactive protein values. The ROC-Curve test (p<0.05)

Variable	Area	p	»Cut-off«
C-reactive protein (mg/L)	0.877	0.001	55.00
Erythrocyte sedimentation rate (mm/hour)	0.645	0.191	–

The correlation tests which were performed for each group data separately revealed no correlation among the parameters. On the other hand, the correlation test applied to the results of all patients, a positive correlation was found between GOS scores and hemoglobin levels (r = 0.445, p = 0.016) and between ESR and CRP values (r = 0.457, p = 0.013). In addition, a negative correlation was found between GOS scores and ESR levels (r = -0.369, p = 0.049), between the GOS scores and the duration of stay in hospital (r = -0.412, p = 0.026) and between ESR and hemoglobin levels (r = -0.399, p = 032). The *Likelihood Ratio* test revealed that ESR value (X^2^ = 10.917, p = 0.012) and duration of stay in hospital (X^2^ = 25.102, p < 0.001) could be the best parameters in predicting the short-term prognosis of the patients with solitary brain tumor.

## Discussion

Studies have reported that immune cells consisted of macrophages, neutrophils, and lymphocytes can highly infiltrate in the most of tumors [11]. In these studies, it has been suggested that tumor-associated inflammation is characterized by the infiltration of immune cells such as microglia/macrophages and the production of cytokines and chemokines, and CRP reflects the systemic response to this inflammation in cancer progression [12] [13]. Epidemiological studies have suggested that elevated CRP levels in various cancers may be associated with poor prognosis. Therefore, many authors concluded that CRP may be a prognostic biomarker for many types of cancer [8] [14] [13] [15] [16].

On the other hand, it was reported that an increased inflammatory response was commonly found in brain tumors and it was argued that this response could be associated with brain tumor development and progression [17]. However, it has been shown that glioblastoma cause deterioration of the blood brain barrier, so that circulating inflammatory cells, normally not found in the central nervous system, can reach tumor areas and even tumor cells can secrete some cytokines which can modulate stromal cells activity. But the role in pathophysiology of this tumor has not been investigated yet [8] [18] [19] [20]. In addition, it has been shown that CRP levels may be elevated in the serum of the patients with glioblastoma and serum CRP may be a poor predictive biomarker of prognosis in glioblastoma, independently [8]. On the other hand, CRP was also found with very high amounts in glioblastoma tumor tissue and it did not change the serum CRP level, and that CRP was thought to be of tumor origin. But, it has been concluded that this protein has no modulatory effect on tumor cells, microglia or endothelial cells. On the other hand, it was considered that CRP could be associated with the immunity which shifted to the paths of apoptosis and necrosis and this immunity could cause to the formation of Th2 mediated response in patients with glioblastoma [7] [21]. However, many other studies showed that CRP does not associate with prognosis in brain tumor patients [16]. Moreover, Heikkilä et al. [22] did not found any relationship between the serum CRP and cancer diagnosis and etiology. In addition to CRP, it has been shown that ESR has a prognostic value in treatment of some specific neoplastic diseases and it has been concluded that elevated ESR values may associate with poor prognosis in most of the cancers [8].

In present study, serum CRP level in patients with glioblastoma was found to be in its laboratory reference interval, but it was found to be significantly elevated in patients who had solitary intracranial metastasis. However, despite the elevation of CRP in serum of the patients with metastatic brain tumor, no increase was observed in counts of the blood leukocyte, lymphocyte, neutrophil, monocyte, basophil and eosinophile in both groups. With these findings, it was thought that this elevation of CRP might be a marker of other immunity pathways (such as apoptosis, autophagy or necrosis) rather than inflammatory cellular response [7] [16] [21]. Besides this, it was thought that this elevated level of serum CRP in patient with metastatic tumor itself. However, because of retrospective nature of this study, the molecular analysis methods which can support these ideas mentioned above could not be applied and discussed in this study. On the other hand, the ROC-curve test results demonstrated that the intracranial tumor could have metastatic origin with high possibility, when the serum CRP was measured > 55.00 mg/L. The regression test results supported that this parameter could be the best parameter distinguishing the metastatic brain tumor from glioblastoma in patient with the solitary intracranial tumor. Therefore, these findings suggested that CRP could be a strong predictive biomarker that could separate intracranial solitary metastasis from glioblastoma.

In literature, it has been reported that ESR may be a prognostic biomarker for patients with glioblastoma or extracranial tumors [8]. In present study, ESR values were not different between the groups and they were within normal reference range in patients with glioblastoma, whereas they were numerically higher in patients with brain metastasis. Besides this, the correlation tests which were separately performed for each group data revealed no correlation among the parameters. Therefore, neither ESR values nor other biochemical findings or demographic data were considered to be a prognostic biomarker predicting the short-term prognosis in either patients with glioblastoma or patients with solitary brain metastasis.

On the other hand, at the end of the correlation test applied to the results of all patients, regardless of patient groups, GOS scores were negatively correlated with the ESR levels and duration of stay in hospital. It could be thougth with these finding that the shortterm prognosis of these patients could be deteriorated if the ESR values of the patients were measured high and/or they have prolonged hospital stay. Therefore, it could be argued that ESR could be a prognostic biomarker for patients with solitary intracranial tumor. In addition, ESR values were positively correlated with the CRP values and it was thought that ESR levels could increase due to the increased CRP. Therefore, it was predicted that the patient's short-term prognosis could be indirectly affected from this increase of CRP. Thus, it was thought that CRP level might be an indirect prognostic biomarker predicting the short-term prognosis in patients with solitary brain tumor (either glioblastoma or metastatic tumor). In addition, GOS scores were positively correlated with hemoglobin levels and it was considered with this finding that GOS scores could be significantly decreased if the hemoglobin level was found to be low in patients with solitary brain tumor. Therefore, it was thought that the hemoglobin levels had a direct effect on short-term prognosis of these patients and this parameter could be used as a prognostic biomarker. In summary, it was thought that hemoglobin and ESR levels measured preoperatively in patients with solitary brain tumor (either glioblastoma or metastatic tumor) may be direct prognostic biomarkers, and CRP levels may be indirect prognostic biomarkers. However, the regression tests results revealed that only ESR value and duration of the stay in hospital could be the best prognostic biomarkers in patients with solitary brain tumor.

### Limitations

There were some shortcomings in this study. *Firstly*, although the number of patients was very small in each group, it was concluded that this study could be a preliminary study because of its quite notable results. Therefore, similar studies on larger series are needed to provide information for the literature in terms of results. *Secondly*, it was concluded that the high CRP levels detected in this study were independent from the cellular inflammatory response, and this was thought to be a marker of other immunity pathways (such as apoptosis, autophagy, necrosis). However, molecular analysis methods which could support this idea were not used in this study because of retrospective property of this study. Therefore, it could be strongly suggested to be supported and explained this idea by more advanced studies with large number of patients. *Thirdly*, long-term follow-up data of the participants was not included in this study due to the retrospective properties of this study. *Finally*, the data of the radiological images of the patients were not evaluated because the objective of this study was to identify the predictive and prognostic biomarkers derived from routine biochemistry tests in patients with solitary intracranial tumor.

## Conclusion

At the end of this study, it was considered that CRP level values measured preoperatively in blood serum of patients with solitary intracranial tumor could be a strong predictive biomarker in distinguishing metastatic brain tumor from glioblastoma. In addition, it was concluded that ESR, CRP, blood hemoglobin levels and duration of stay in hospital could be biomarkers in predicting the short-term prognosis of patients with solitary brain tumor (either glioblastoma or metastatic tumor). However, it was considered that these findings should be supported and explained by more advanced studies consisted of large number of patients.

## Acknowledgements

The authors would like to thank Mikail Inal, MD, Associate Professor, for his invaluable assistance in the interpretation of the radiological images in this study.

## Conflict of interest statement

All the authors declare that they have no conflict of interest in this work.

## List of abbreviations

ALT, alanine aminotransferase; AST, aspartate aminotransferase; BUN, blood urea nitrogen; CRP, C-reactive protein; CT, computerized tomography; ESR, erythrocyte sedimentation rate; GCS, Glasgow Coma Scale; GOS, Glasgow Outcome Scale; Hb, hemoglobin; K, potassium; MR, magneticresonance; Na, sodium; NLR, neutrophil to lymphocyte ratio; PLR, platelet to lymphocyte ratio.
